# Discoveries Interview: Professor Bhanu P. Jena on the discovery of the porosome, the universal machinery for cellular secretion

**DOI:** 10.15190/d.2014.20

**Published:** 2014-09-08

**Figure 1:**
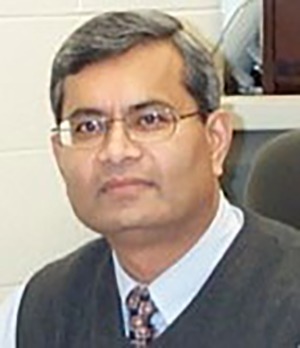
Professor Bhanu P. Jena

**Professor Bhanu P. Jena** received his Ph.D. degree in Endocrinology, and the Research Excellence Award from Iowa State University in 1988. Following post-doctoral training at Iowa State and Yale Universities (1989-1994), Prof. Jena joined Yale University, as an Assistant Professor, and in 2000, moved to the Department of Physiology, at Wayne State University School of Medicine, as Professor, and Founder-Director of the Institute of NanoBioScience. In 2004, he was conferred the distinction of George E. Palade University Professor and Distinguished Professor. Prof. Jena is the only living University Professor, and the second in Wayne State University’s 160-year history, conferred by the board of governors and former Wayne State University’s President, Dr. Irvin D. Reid. At a very early age, Prof. Jena was fascinated by the complexity of the cell, similar to the complexity of a city, yet every aspect of its operation precisely organized and coordinated. His scientific enquiry on how cells secrete which began more than 40 years ago, led to the discovery of the “porosome” -a new cellular structure and the universal secretory portal in cells.

* Prof. Jena* has received numerous awards and honors for his discoveries, among them: Elected Foreign Member of the Georgian National Academy of Science; Fellow AAAS; the Swebelius Cancer Research Award, the Hallim Distinguished Award Lecture jointly with Prof. Ahmed H. Zewail; Sir. Aaron Klug Award; ASAS Basic Biological Science Award; Ranbaxy Basic Research in Medical Sciences Award; Elected Foreign Member of the Korea Academy of Science & Technology; Elected Foreign Member of the National Academy of Medicine, Romania; George E. Palade Gold Medal, elected to the Academy of Scholars at Wayne State University; six Honorary Doctorates including one from Babes-Bolyai University, Romania, jointly with Professors George E. Palade and Günter Blobel, and Distinguished Visiting Professorships from a number of academic institutions.

## 1. Can you define in simple words what is the porosome and why it is important?

Porosomes are secretory portals at the cell plasma membrane. They are cup-shaped lipoprotein structures, where secretory vesicles transiently dock and fuse to expel intra-vesicular contents from cells during secretion. Porosomes range in size from 15 nm in neurons and astrocytes to 100-180 nm in the exocrine pancreas and neuroendocrine cells. Porosomes have been isolated from a number of cells, and their morphology, composition, and functional reconstitution well documented^1-4^. The 3D contour map of the assembly of proteins within the porosome complex, and its native X-ray solution structure at sub-nm resolution has also been determined^5^. Porosomes are composed of nearly 30 proteins^6^. In comparison, the nuclear pore complex measures 120 nm and is comprised of over 500 protein molecules.

It was believed that during cell secretion, membrane-bound secretory vesicles completely collapse at the cell plasma membrane, resulting in the diffusion of intra-vesicular contents to the outside and the compensatory retrieval of the excess membrane by endocytosis. Complete vesicle merger however fails to explain the generation of partially empty vesicles observed in electron micrographs in cells following secretion, suggesting the involvement of an additional mechanism that would enable the release of a portion of the vesicle content. The partial emptying of vesicles during secretion occurs when secretory vesicles temporarily establish continuity with the cell plasma membrane at the porosome base via a fusion pore^1-5^, expell a portion of vesicle contents resulting from the regulated differential swelling of secretory vesicles^7-9^. The partially empty secretory vesicle can then detach, reseal, and withdraw into the cytosol by endocytosis. Utilizing this mechanism, secretion could be precisely regulated and secretory vesicle could be reused for subsequent rounds of exo-endocytosis, until completely empty of contents.

## 2. How was porosome discovered and how our knowledge about it evolved over time?

In the mid 1996, employing the then newly developed technique of AFM, nanometer scale pore structures and their dynamics were discovered at the apical plasma membrane in live pancreatic acinar cells. Circular pit-like structures containing 100-180 nm cup-shaped depressions or pores were observed at the apical plasma membrane of pancreatic acinar cells where secretion is known to occur^1^. During secretion, the depression or pore opening grew larger, returning to its resting size following completion of cell secretion. Then in 2002 and 2003, studies established the observed depressions to be secretory portals at the cell plasma membrane^10,11^. In 2002^12^, it was demonstrated that following stimulation of cell secretion, gold-conjugated amylase antibodies (amylase being one of the major intra-vesicular enzymes secreted by pancreatic acinar cells) accumulate at depressions. These results established depressions to be the long sought-after secretory portals^13,14^ in cells. The study further reported^10^ the presence of t-SNAREs at the porosome base facing the cytosol, firmly establishing depression structures to be secretory portals where vesicles transiently dock and fuse for intra-vesicular content release during secretion^10^. Subsequently depressions and their dynamics at the cell plasma membrane in chromaffin cells^15^, and in growth hormone (GH) secreting cells of the pituitary gland^16^, were reported. In 2003, following immunoisolation of the porosome from acinar cells of the exocrine pancreas, their composition was determined, and they were both structurally and functionally reconstituted into artificial lipid membranes^11^. Morphological details of porosome structures in association with docked secretory vesicles have also been elucidated using high-resolution EM^5,11^. In the past decade, employing a combination of approaches such as AFM, biochemistry, electrophysiology, conventional EM, mass spectrometry, and small angle X-Ray solution scattering (SAXS) analysis, this specialized portal has been found to be present in all secretory cells examined, including neurons^6,7^. Consequently, these structures have been named ‘porosomes’ or secretory portals in cells.

## 3. How our knowledge on porosome will help understand and target human diseases? What will the field look like in 5-10 years?

Secretion is a fundamental cellular process that occurs in every organism, from the yeast to humans. For example, secretion of neurotransmitters at the nerve terminal enable neurotransmission, allowing thought, movement, and coordination. Similarly after a meal, secretion of digestive enzymes from the exocrine pancreas help digest food. The consequent elevation of blood glucose following digestion, triggers secretion of insulin from β-cells of the endocrine pancreas. Similarly, exposure to certain types of pollen, or to a parasite, elicits an allergic inflammatory immune response, stimulating mast cells to secrete histamine and other compounds. Hence, a molecular understanding of cell secretion has profound implications in the understanding and targeting of disease in humans resulting from secretory defects. Let’s take the example of cystic fibrosis (CF), the disease. CF is caused by the malfunction of CF transmembrane conductance regulator (CFTR), a chloride channel transporter, resulting in viscous mucus in the airways. Studies in mice lacking functional CFTR secrete highly viscous mucous that adhered to the epithelium. Recent studies using the human airways epithelia cell line Calu-3, demonstrate the presence of mucin-secreting porosomes and the association of CFTR with the complex. These new findings will facilitate understanding of CFTR-porosome interactions influencing mucus secretion, and provide critical insights into the etiology of CF disease.

Unlike individual proteins or lipids, determination of the atomic structure of such dynamic macromolecular lipoprotein complexes such as the porosome, poses a difficult challenge, requiring the use of several experimental and computational approaches to maximize resolution and accuracy. The next 5-10 years will see the resolution of the porosome structure at the atomic level.

## 4. What advices do you have for young scientists?

Understanding how nature works is the most rewarding experience for a scientist. Simple child-like curiosity is the best approach for doing science.

## 5. In your opinion, what are the most challenging, promising and/or the most rewarding areas of research?

Research to elucidate all fundamental aspects of nature is exciting and rewarding. It is what aspects of how nature works excites you. To me, understanding the structure and dynamics of a live cell at the molecular level, would be most exciting and rewarding.
